# Psychological Impairment and Coping Strategies During the COVID-19 Pandemic Among Students in Pakistan: A Cross-Sectional Analysis

**DOI:** 10.1017/dmp.2020.397

**Published:** 2020-10-22

**Authors:** Muhammad Salman, Noman Asif, Zia Ul Mustafa, Tahir Mehmood Khan, Naureen Shehzadi, Humera Tahir, Muhammad Husnnain Raza, Muhammad Tanveer Khan, Khalid Hussain, Yusra Habib Khan, Muhammad Hammad Butt, Tauqeer Hussain Mallhi

**Affiliations:** Faculty of Pharmacy, The University of Lahore, 1-Km Defense road, Lahore, Pakistan; Punjab University College of Pharmacy, University of the Punjab, Lahore, Pakistan; Gulab Devi Educational Complex, Lahore, Pakistan; Department of Pharmacy Services, District Headquarter Hospital, Pakpattan, Pakistan; Institute of Pharmaceutical Science, University of Veterinary and Animal Sciences, Lahore, Pakistan; School of Pharmacy, Monash University, Bandar Sunway, Selangor 45700 Malaysia; Ruth Pfau College of Nutrition Sciences, Lahore Medical and Dental College, Lahore, Pakistan; Faisalabad Institute of Cardiology, Faisalabad, Pakistan; Department of Clinical Pharmacy, College of Pharmacy, Jouf University, Sakaka, Al-Jouf, Kingdom of Saudi Arabia; Faculty of Pharmacy, University of Central Punjab, Lahore, Pakistan

**Keywords:** anxiety, coping, COVID-19, depression, Pakistan, university students

## Abstract

High levels of stress are expected when crises affect people’s lives. Therefore, this Web-based, cross-sectional study was conducted among university students from Pakistan to investigate the psychological impairment and coping strategies during the coronavirus disease 2019 (COVID-19) pandemic. Google Forms were used to disseminate the online questionnaire to assess anxiety (Generalized Anxiety Disorder-7), depression (Patient Health Questionnaire-9), and coping strategies (Brief-COPE). A total of 1134 responses (age, 21.7 ± 3.5 y) were included. The frequency of students having moderate-severe anxiety and depression (score ≥ 10) were ≈ 34% and 45%, respectively. The respondents’ aged ≥ 31 y had significantly lower depression score than those ≤ 20 y (*P* = 0.047). Males had significantly less anxiety (6.62 ± 5.70 vs 7.84 ± 5.60; *P* = 0.001) and depression (8.73 ± 6.84 vs 9.71 ± 7.06; *P* = 0.031) scores. Those having family members, friends, or acquaintances infected with disease had significantly higher anxiety scores (8.89 ± 5.74 vs 7.09 ± 5.56; *P* < 0.001). Regarding coping strategies, the majority of respondents were found to have adopted religious/spiritual coping (6.45 ± 1.68) followed by acceptance (5.58 ± 1.65), self-distraction (4.97 ± 1.61), and active coping (4.81 ± 1.57). In conclusion, COVID-19 caused significant impairment on mental health of the students. The most frequent coping strategies adopted by students were religious/spiritual and acceptance coping. During epidemics, mental health of students should not be neglected.

Historically, emergence and re-emergence of large-scale infectious diseases (IDs) have had civilization-altering consequences. In addition to physical problems, a variety of psychosocial problems also emerge, mainly due to the lack of sufficient knowledge about these IDs. Initially, fear, anxiety, and hysteria among people are observed that lead to stigma, irrational response to the disease, in the society. Recent examples in this context include stigmatization of HIV/AIDS, severe acute respiratory syndrome (SARS), H1N1, and Ebola. Such impulsive reactions reveal the enormous psychological distress consequential of emerging diseases, particularly when it is unfamiliar, highly contagious, and fatal, such as this ongoing coronavirus disease 2019 (COVID-19) pandemic.^1^

In Pakistan, the first case of COVID-19 appeared on February 26, 2020. The situation escalated quickly, and a complete lock-down was imposed in the country on March 23, 2020, to effectively contain COVID-19. This complete lock-down was converted into “smart lockdown” on May 9, 2020. However, all the education institutions as well as big markets and all public places were directed to remain closed.^2,3^

The continuous spread of the disease, conspiracy theories, myths, and blame games; sensational media reporting of COVID-19; frustration and boredom; implementation of social lock-down with classmates, friends, and teachers; lack of personal space at home; and family financial loss due to lock-down are some of the main risk factors significantly influencing the mental health of the university students. There have been reports on the psychological impact of the epidemic on the general public, health-care workers, and college students.^3-7^ However, to the best of our knowledge, no studies have assessed the psychological impairment of COVID-19 on Pakistani university students. Therefore, the present study was conducted to underscore the psychologic impact of CVOID-19 on Pakistani university students and their coping strategies.

## METHODS

### Study Design, Settings, and Subjects

A Web-based, cross-sectional survey was conducted among Pakistani university students in the months of April and May 2020 during the complete lockdown in Pakistan. All the students acquiring education at the University of the Punjab, The University of Lahore, Gulab Devi Educational Complex, and University of Veterinary and Animal Sciences were eligible for inclusion in this study. We excluded those who were not university students, who were already graduated, and those unwilling to take part in the study.

### Ethical Considerations

Protocol of the present study was reviewed and approved by the Research Ethics Committee of the Department of Pharmacy Practice, Faculty of Pharmacy, The University of Lahore (REC/DPP/FOP/16). An informed consent was obtained from every study participant.

### Data Collection Tool

Google Forms were used to disseminate the online questionnaire among the students, aiming to assess anxiety, depression, and the coping strategies during the ongoing COVID-19 pandemic. The contents of the questionnaire were reviewed by an expert panel and, after suggested changes have been made, were approved for data collection. Additionally, the questionnaire was piloted among 10 Doctor of Pharmacy students at the University of Lahore (age, 20-30 y; 4 males and 6 females). All the participants reported ease of understanding all the items and their response options; content validity index reached 1 for all the scales. Data of these participants were not included in the final study.

### Outcome Measure

In the present study, Generalized Anxiety Disorder scale (GAD-7) was used to assess anxiety.^8^ It contains 7 items, each of which is scored 0 (not at all) to 3 (nearly every day), providing a 0 to 21 score. Scores of 5-9, 10-14, and ≥ 15 are taken as the cutoff points for mild, moderate, and severe anxiety, respectively. Using a cutoff score of ≥ 10, the GAD-7 has a sensitivity of 89% and a specificity of 82%. Moreover, it is also moderately good at screening 3 other common anxiety disorders: panic disorder (sensitivity 74% and specificity 81%), social anxiety disorder (sensitivity 72% and specificity 80%), and posttraumatic stress disorder (sensitivity 66% and specificity 81%).

Patient Health Questionnaire (PHQ-9) was used to screen depression in the study participants. PHQ-9 is one of the most commonly used instruments in practice as well as research. It contains 9-items each of which is scored 0 (not at all) to 3 (nearly every day), yielding a 0 to 27 score. PHQ-9 scores of ≤ 4, 5-9, 10-14, 15-19, and ≥ 20 are considered minimal, mild, moderate, moderately severe, and severe depression, respectively.^9^ The sources of distress from the ongoing COVID-19 epidemic were enquired with 14 questions formed by the researchers based on a previous study reporting anxiety among university students during the SARS outbreak.^10^

The Brief-COPE questionnaire was used to evaluate the coping strategies adopted by the study participants. It is a validated 28-item self-report questionnaire that measures effective and ineffective ways to cope with a stressful life event.^11^ Responses to each item are scored from 1 (I have not been doing this at all) to 4 (I have been doing this a lot). The Brief-Cope explores the following 14 coping methods: self-distraction, active coping, denial, substance use, use of emotional support, use of instrumental support, behavioral disengagement, venting, positive reframing, planning, humor, acceptance, religion, and self-blame. Possible scores ranged from 2 to 8 for each coping style; higher scores indicated a higher tendency to implement the corresponding coping style.

The previously reported prevalence rate of depression, stress, and anxiety among students comes out as 48.0%, 53.2%, and 68.54%, respectively^12^

### Data Analysis

Responses stored in the Web-based database (The Google Drive) of the principal investigator were transferred to Microsoft Excel sheet. After appropriate coding and data cleaning, the data were imported into the SPSS version 22 for the analysis. Continuous variables were expressed as mean and standard deviations (SD), whereas frequency and percentages were used to present categorical data. Independent sample t-test was applied to estimate the difference among 2 groups, whereas 1-way analysis of variance (ANOVA) was applied for 3 or more groups. Furthermore, Tukey’s HSD and Games-Howell post hoc tests were performed to assess significance among intergroup variables, where applicable. A *P* value of less than 0.05 was taken for statistical significance.

## RESULTS

### Characteristics of the Study Sample

A total of 1134 responses were included in this study. Upon analysis, it was revealed that a majority (70.5%) of the respondents were female and native of Punjab province (93.4%), and 67.9% of the students were enrolled in the Doctor of Pharmacy program. Approximately 22% of the respondents disclosed having a family member, relative, friend, or acquaintance infected with the disease.

### Anxiety and Depression Among the Respondents

The mean anxiety and depression scores were 7.48 ± 5.65 and 9.42 ± 7.01, respectively. The frequency of students having moderate to severe anxiety (score ≥ 10) was ≈ 34%. Regarding the severity of depression, 30.5%, 24.5%, 21%, 13.6%, and 10.4% students were found to have minimal-none, mild, moderate, moderately severe, and severe depression, respectively. Between-demographic analysis of anxiety and depression scores are described in Table 1. Initially, a significant difference of depression scores, not the anxiety scores (*P* = 0.658), was observed between age groups (*P* = 0.048). However, in multiple comparisons using Tukey’s honestly significant difference (HSD) test (Table 2), only the respondents aged 31 y and above had significantly lower depression scores than those below or equal to 20 y of age (*P* = 0.047). Similarly, male respondents were observed to have significantly less anxiety (*P* = 0.001) and depression (*P* = 0.031) than female respondents. In addition, those who reported having family members, relatives, friends, or acquaintances who contracted the disease had a significantly higher anxiety score (*P* < 0.001).

**TABLE 1:** Anxiety and Depression Assessment Based on Demographics of Respondents

Demographics	Subgroups	*N* (%)	Anxiety Score	Depression Score
Age	≤ 20 y	462 (40.7%)	7.38 ± 5.57	9.82 ± 6.95
	> 20-25 y	574 (50.6%)	7.56 ± 5.72	9.35 ± 7.02
	> 25-30 y	66 (5.8%)	7.94 ± 5.72	8.64 ± 7.41
	≥ 31 y	32 (2.8%)	6.53 ± 5.55	6.50 ± 6.18*
	F value (*P*-value)	--	0.536 (0.658)	2.650 (0.048)
Gender	Male	335 (29.5%)	6.62 ± 5.70	8.73 ± 6.84
	Female	799 (70.5%)	7.84 ± 5.60	9.71 ± 7.06
	T value (*P*-value)	--	−3.337 (0.001)	−2.159 (0.031)
Province	Punjab	1059 (93.4%)	7.42 ± 5.63	9.35 ± 6.93
	Other provinces	75 (6.6%)	8.11 ± 5.89	10.17 ± 7.91
	T value (*P*-value)	−	−1.011 (0.312)	−0.98 (0.327)
Education	Medical	43 (3.8%)	7.19 ± 5.67	7.74 ± 6.59
	Pharmacy	770 (67.9%)	7.48 ± 5.74	9.52 ± 6.99
	Allied Health Sciences	62 (5.5%)	7.19 ± 5.37	8.32 ± 6.27
	Others	259 (22.8%)	7.60 ± 5.47	9.67 ± 7.27
	F value (*P*-value)	−	0.130 (0.943)	1.485 (0.217)
Family member, relative, or acquaintance got COVID-19	Yes	247(21.8%)	8.89 ± 5.74	10.06 ± 6.91
	No	887 (78.2%)	7.09 ± 5.56	9.24 ± 7.03
	T value (*P*-value)	−	4.464 (< 0.001)	0.543 (0.101)

**TABLE 2:** Post-Hoc Analysis of Depression Scores Among Age Groups

Multiple Comparisons	Mean Differences	SE	*P*-Value*	95% CI	
				Lower Bound	Upper Bound
≤ 20 y vs > 20-25 y	0.466	0.437	0.710	−0.66	1.59
≤ 20 y vs > 25-30 y	1.180	0.920	0.574	−1.19	3.55
≤ 20 y vs ≥ 31 y	3.316	1.278	0.047	0.03	6.60
> 20-25 y vs > 25-30 y	0.714	0.909	0.861	−1.62	3.05
> 20-25 y vs ≥ 31 y	2.850	1.270	0.112	−0.42	6.12
>25-30 y vs ≥ 31 y	2.136	1.506	0.488	−1.74	6.01

* Tukey’s HSD test.

### Sources of Distress

Major distresses were related to the effects of the COVID-19 pandemic on the routine life of students. The majority of study participants reported that COVID-19 has turned their lives upside down (80.8%) and restricted social meetings with friends (84.7%), shopping, sporting, and other important activities (88.3%). Approximately 80% reported being afraid of travelling in transport with air-conditioning. Regarding the fear of health of self and family members, the majority (70.9%) of respondents expressed fear of their family members and friends getting infected. However, approximately 41% were afraid they could contract the disease, and 34.9% reported that sometimes they suspect that they have been infected. Nearly 71% were scared of visiting health-care settings due to the fear of the disease.

Regarding the SARS coronavirus 2 (SARS-CoV2) spread in Pakistan, 78.2% of respondents reported that the virus was spreading at an alarming rate, 78.3% were afraid it would be immensely difficult to control in Pakistan, and 62.3% were afraid it would persist in the community for a very long time. In the present study, approximately 60% respondents believed that the authorities were not revealing enough, adequate, and true information; 58.2% were stressed because large-scale COVID-19 testing was not being done; and 68.1% reported that those suffering from the disease were not receiving good medical care.

### Coping Strategies of the Study Participants

The overall Brief-COPE score of the respondents was 57.22 ± 12.29. As shown in Table 3, mean score was higher for religious coping (6.45 ± 1.68) followed by acceptance (5.58 ± 1.65), whereas it was the lowest for substance use (1.85 ± 1.35). Regarding the intra-demographic differences of coping strategies, there was no significant difference of the coping styles among age categories except for active coping, substance use, positive reframing, planning, and self-blame. However, in multiple comparisons, no significant difference was found for active coping, substance use, and positive reframing scores. The students belonging to the ≤ 20 y age group had significantly lower scores for coping planning than those from the 26-30 and ≥ 31 y age groups (Table 4). Moreover, the students belonging to the 21-25 y age group had significantly higher self-blame scores than those from the 26-30 and ≥ 31 y age groups. Gender was observed to be 1 of the main factors where significant differences were observed for self-distraction, planning, humor, acceptance, and religious coping. Male respondents were observed to have significantly lower scores for the self-distraction (*P* < 0.001), acceptance (*P* = 0.019), and religious coping (*P* < 0.001), while females had lower scores for planning (*P* = 0.033) and humor coping (*P* < 0.001). Among education categories (Table 4), medical students had significantly less “self-blame” score than pharmacy (*P* = 0.005), allied health sciences (*P* = 0.028), and other university students (*P* = 0.002). Moreover, mean scores of acceptance coping were significantly higher among those having family members, relatives, friends, or acquaintance infected with COVID-19 than the ones who had not (*P* = 0.026).

**TABLE 3:** Coping Strategies Adopted by the Study Participants

Variable	Subgroups	Self-Distraction	Active Coping	Denial	Substance Use	Emotional Support	Informational Support	Behavioral Disengage-ent	Venting	Positive Reframing	Planning	Humor	Acceptance	Religion	Self-blame
Overall	−	4.97 ± 1.61	4.81 ± 1.57	3.30 ± 1.59	1.85 ± 1.35	4.02 ± 1.67	3.62 ± 1.79	3.53 ± 1.54	3.79 ± 1.53	4.73 ± 1.71	4.63 ± 1.67	2.85 ± 1.40	5.58 ± 1.65	6.45 ± 1.68	3.11 ± 1.61
Age	≤ 20 y	4.96 ± 1.60	4.66 ± 1.47	3.21 ± 1.60	1.78 ± 1.23	3.97 ± 1.63	3.60 ± 1.75	3.49 ± 1.50	3.74 ± 1.55	4.56 ± 1.62	4.43 ± 1.63	2.77 ± 1.35	5.49 ± 1.67	6.48 ± 1.65	3.07 ± 1.59
	> 20-25 y	5.02 ± 1.63	4.89 ± 1.60	3.32 ± 1.60	1.95 ± 1.47	4.02 ± 1.69	3.62 ± 1.84	3.58 ± 1.58	3.83 ± 1.52	4.81 ± 1.77	4.69 ± 1.68	2.92 ± 1.46	5.59 ± 1.63	6.38 ± 1.72	3.23 ± 1.69
	> 25-30 y	4.72 ± 1.63	5.14 ± 1.76	3.52 ± 1.56	1.58 ± 1.01	3.91 ± 1.59	3.60 ± 1.75	3.34 ± 1.52	3.89 ± 1.50	5.12 ± 1.84	5.09 ± 1.76	2.74 ± 1.24	5.86 ± 1.75	6.43 ± 1.67	2.71 ± 1.23
	≥ 31 y	4.53 ± 1.39	4.81 ± 1.67	3.75 ± 1.39	1.63 ± 1.16	4.78 ± 1.81	4.03 ± 1.77	3.22 ± 1.26	3.56 ± 1.27	4.91 ± 1.38	5.38 ± 1.64	3.00 ± 1.32	5.78 ± 1.66	6.94 ± 1.32	2.66 ± 1.10
	F value (*P*-value)	1.517 (0.208)	2.843 (0.037)	1.798 (0.146)	3.059 (0.031)*	2.469 (0.061)	0.577 (0.630)	1.101 (0.347)	0.619 (0.603)	3.186 (0.027)*	6.248 (< 0.001)	1.244 (0.297)*	1.194 (0.311)	1.821 (0.147)*	5.046 (0.003)*
Gender	Male	4.68 ±1.57	4.70 ± 1.63	3.43 ± 1.58	1.95 ± 1.44	3.99 ± 1.62	3.65 ± 1.82	3.59 ± 1.56	3.74 ± 1.51	4.58 ± 1.75	4.79 ± 1.78	3.24 ± 1.61	5.40 ± 1.75	6.13 ± 1.84	3.20 ± 1.63
	Female	5.09 ± 1.62	4.86 ± 1.53	3.25 ± 1.60	1.81 ± 1.31	4.03 ± 1.69	3.61 ± 1.78	3.49 ± 1.53	3.81 ± 1.53	4.80 ± 1.68	4.55 ± 1.63	2.68 ± 1.27	5.65 ± 1.61	6.57 ± 1.59	3.09 ± 1.61
	T value (*P*-value)	−3.96 (< 0.001)	−1.558 (0.119)	1.779 (0.075)	1.546 (0.123)	−0.299 (0.765)	0.308 (0.758)	0.947 (0.344)	−0.780 (0.436)	−1.965 (0.050)	2.130 (0.033)	5.648 (< 0.001)	−2.339 (0.019)	−3.810 (< 0.001)	1.035 (0.301)
Province	Punjab	4.96 ± 1.61	4.80 ± 1.55	3.29 ± 1.58	1.87 ± 1.35	4.02 ± 1.66	3.61 ± 1.78	3.53 ± 1.53	3.79 ± 1.51	4.73 ± 1.70	4.60 ± 1.66	2.83 ± 1.37	5.56 ± 1.65	6.42 ± 1.68	3.12 ± 1.60
	Other provinces	5.04 ± 1.54	4.93 ± 1.79	3.55 ± 1.75	1.61 ± 1.26	4.01 ± 1.87	3.72 ± 2.01	3.33 ± 1.67	3.78 ± 1.65	4.78 ± 1.87	4.97 ± 1.83	3.19 ± 1.76	5.72 ± 1.76	6.67 ± 1.70	3.20 ± 1.88
	T value (*P*-value)	−0.411 (0.681)	−0.636 (0.525)	−1.338 (0.181)	1.533 (0.126)	0.022 (0.983	−0.452 (0.653)	1.049 (0.295)	0.042 (0.976)	−0.253 (0.800)	−1.790 (0.074)	−1.680 (0.097)	−0.800 (0.424)	−1.172 (0.241)	−0.372 (0.711)
Education	Medical	4.77 ± 1.41	4.63 ± 1.56	2.84 ± 1.05	1.70 ± 1.41	3.91 ± 1.67	3.21 ± 1.85	3.07 ± 1.37	3.65 ± 1.60	4.33 ± 1.58	4.44 ± 1.45	3.14 ± 1.54	5.58 ± 1.68	6.09 ± 1.95	2.51 ± 1.03
	Pharmacy	4.98 ± 1.61	4.82 ± 1.56	3.35 ± 1.60	1.91 ± 1.37	4.01 ± 1.65	3.68 ± 1.79	3.54 ± 1.55	3.81 ± 1.55	4.76 ± 1.71	4.65 ± 1.70	2.78 ± 1.35	5.55 ± 1.66	6.46 ± 1.68	3.10 ± 1.60
		5.10 ± 1.68	4.85 ± 1.60	3.10 ± 1.50	1.76 ± 1.48	3.92 ± 1.43	3.66 ± 1.65	3.53 ± 1.47	3.79 ± 1.48	4.76 ± 1.60	4.71 ± 1.71	2.98 ± 1.53	5.35 ± 1.60	6.26 ± 1.74	3.29 ± 1.78
	Allied Health Sciences Others	4.94 ± 1.64	4.81 ± 1.58	3.28 ± 1.65	1.71 ± 1.22	4.08 ± 1.77	3.51 ± 1.83	3.54 ± 1.54	3.76 ± 1.45	4.71 ± 1.74	4.56 ± 1.65	2.97 ± 1.49	5.68 ± 1.67	6.47 ± 1.62	3.23 ± 1.69
	F value (*P*-value)	0.395 (0.757)	0.219 (0.883)	3.363 (0.021)*	1.735 (0.158)	0.251 (0.861)	1.375 (0.249)	1.279 (0.280)	0.187 (0.905)	0.906 (0.437)	0.411 (0.745)	2.141 (0.093)	0.749 (0.523)	0.922 (0.429)	5.348 (0.002)*
Family member, relative, or acquaintance got COVID-19	Yes	5.04 ± 1.65	4.84 ± 1.56	3.14 ± 1.49	1.99 ± 1.52	4.09 ± 1.75	3.69 ± 1.87	3.56 ± 1.58	3.84 ± 1.45	4.78 ± 1.76	4.63 ± 1.66	2.85 ± 1.39	5.78 ± 1.58	6.41 ± 1.65	3.24 ± 1.65
	No	4.95 ± 1.60	4.81 ± 1.57	3.34 ± 1.62	1.81 ± 1.29	4.00 ± 1.65	3.60 ± 1.77	3.51 ± 1.53	3.78 ± 1.55	4.72 ± 1.69	4.62 ± 1.68	2.85 ± 1.40	5.52 ± 1.67	6.45 ± 1.69	3.09 ± 1.60
	T value (*P*-value)	0.753 (0.452)	0.258 (0.796)	−1.742 (0.082)	1.692 (0.091)	0.820 (0.412)	0.687 (0.492)	0.407 (0.684)	0.511 (0.609)	0.519 (0.604)	0.102 (0.918)	0.060 (0.953)	2.232 (0.026)	−0.279 (0.780)	1.329 (0.184)

* Welch’s ANOVA was used instead of classic ANOVA as the assumption of homogeneity of variances was violated.

**TABLE 4:** Multiple Comparisons of Coping Strategies Among Age and Education Variables

Multiple comparisons	Mean Differences	SE	*P*-Value	95% CI	
				Lower Bound	Upper Bound
Age	Active Coping*				
≤ 20 y vs > 20-25 y	−0.227	0.098	0.093	−0.48	0.02
≤ 20 y vs > 25-30 y	−0.439	0.206	0.142	−0.97	0.09
≤ 20 y vs ≥ 31 y	−0.146	0.285	0.957	−0.88	0.59
> 20-25 y vs > 25-30 y	−0.212	0.203	0.722	−0.73	0.31
> 20-25 y vs ≥ 31 y	0.081	0.284	0.992	−0.65	0.81
>25-30 y vs ≥ 31 y	0.294	0.336	0.819	−0.57	1.16
Age	Substance Use*				
≤ 20 y vs > 20-25 y	−0.169	0.084	0.186	−0.39	0.05
≤ 20 y vs > 25-30 y	0.195	0.138	0.495	−0.17	0.56
≤ 20 y vs ≥ 31 y	0.155	0.212	0.885	−0.42	0.73
> 20-25 y vs > 25-30 y	0.364	0.140	0.051	0.00	0.73
> 20-25 y vs ≥ 31 y	0.324	0.214	0.438	−0.25	0.90
>25-30 y vs ≥ 31 y	−0.040	0.240	0.988	−0.68	0.60
Age	Positive Reframing**				
≤ 20 y vs > 20-25 y	−0.252	0.105	0.080	−0.50	0.02
≤ 20 y vs > 25-30 y	−0.509	0.242	0.161	−1.14	0.13
≤ 20 y vs ≥ 31 y	−0.339	0.255	0.549	−1.02	0.35
> 20-25 y vs > 25-30 y	−0.257	0.241	0.712	−0.89	0.38
> 20-25 y vs ≥ 31 y	−0.087	0.254	0.986	−0.77	0.60
>25-30 y vs ≥ 31 y	0.170	0.335	0.957	−0.71	1.05
Age	Planning*				
≤ 20 y vs > 20-25 y	−0.257	0.104--	0.064	−0.52	0.01
≤ 20 y vs > 25-30 y	−0.660	0.219--	0.014	−1.22	−0.10
≤ 20 y vs ≥ 31 y	−0.944	0.304--	0.010	−1.73	−0.16
> 20-25 y vs > 25-30 y	−0.403	0.216	0.244	−0.96	0.15
> 20-25 y vs ≥ 31 y	−0.687	0.302	0.104	−1.46	0.09
>25-30 y vs ≥ 31 y	−0.284	0.358	0.857	−1.20	0.64
Age	Self-Blame**				
≤ 20 y vs > 20-25 y	−0.158	0.102	0.409	−0.42	0.10
≤ 20 y vs > 25-30 y	0.370	0.168	0.130	−0.07	0.81
≤ 20 y vs ≥ 31 y	0.411	0.207	0.211	−0.14	0.97
> 20-25 y vs > 25-30 y	0.528	0.167	0.011	0.09	0.96
> 20-25 y vs ≥ 31 y	0.568	0.206	0.042	0.02	1.12
>25-30 y vs ≥ 31 y	0.041	0.246	0.998	−0.61	0.69
Education	Denial**				
Medical vs Pharmacy	−0.512	0.169	0.019	−0.96	−0.06
Medical vs Allied health sciences	−0.260	0.248	0.724	−0.91	0.39
Medical vs Others	−0.425	0.189	0.119	−0.92	0.07
Pharmacy vs Allied health sciences	0.253	0.199	0.586	−0.27	0.78
Pharmacy vs Others	0.087	0.117	0.881	−0.22	0.39
Allied health sciences vs Others	−0.166	0.216	0.869	−0.73	0.40
Education	Self-Blame**				
Medical vs Pharmacy	−0.586	0.168	0.005	−1.03	−0.14
Medical vs Allied health sciences	−0.779	0.275	0.028	−1.50	−0.06
Medical vs Others	−0.708	0.189	0.002	−1.20	−0.21
Pharmacy vs Allied health sciences	−0.193	0.233	0.841	−0.81	0.42
Pharmacy vs Others	−0.123	0.119	0.733	−0.43	0.19
Allied health sciences vs Others	0.070	0.249	0.992	−0.58	0.72

* Tukey’s HSD test.

** Games Howell post-hoc test.

## DISCUSSION

COVID-19 is the most devastating and challenging public health crises since the influenza pandemic in 1918. As of May 14, 2020, more than 4.2 million people have been infected, and 292,046 succumbed to it globally.^13^ It is undeniable that the disease is causing a great deal of anxiety, fear, and unrest in people of all ages. University students are no exception, as all education institutions are closed in Pakistan and students are uncertain about their academic year (Order No. SO(I&C-I) 1-2/2020). To the best of our knowledge, this is the first study that examines not only the psychological impairment of COVID-19 among university students but also their coping strategies.

Regarding anxiety and depression, a cut-point of ≥ 10 on GAD-7 as well as PHQ-9 is considered a “yellow flag” (drawing attention to a possible clinically relevant condition), while a cut-point of 15 is a “red flag” (individuals in whom active treatment is probably warranted). In the present study, approximately 34% (GAD-7: moderate anxiety = 19.8%; severe anxiety = 13.7%) and 45% (PHQ-9: moderate = 21%; moderately severe = 13.6%; severe depression = 10.4%) of respondents were found to have scores ≥10 on both aforementioned measures, respectively. Contrary to our results, Cao et al. (2020) reported that 21.3%, 2.7%, and 0.9% of Chinese college students had mild, moderate, and severe anxiety, respectively. The significantly higher proportion of students with anxiety and depression in our study can be attributable to the fact that 21.8% of our study subjects having somebody (family members, friends, relatives, neighbors, acquaintances) who have been diagnosed with COVID-19, which was less than 1% in the previous study.^5^ Studies that assessed the psychological impact of SARS and Middle East respiratory syndrome (MERS) coronaviruses outbreaks also found significant impact of these epidemics on mental health of students.^10,14^ Upon enquiring of the impact of anxiety and/or depressive symptoms on the quality of life, 48%, 11.6%, and 6.5% stated somewhat, very, and extreme difficulty in doing work, taking care of things at home, or getting along with others.

Now is the time that academic institutions must work together with the government to promote measures suggested by the World Health Organization to improve the mental health of students during the pandemic. Education authorities should take immediate measures to address the student-related issues. Moreover, students should be encouraged to adopt healthy life style. Educational institutes can engage their students in several online activities, including quizzes, webinars and psychological sessions.^15^

Eisenberg et al. reported 2 major components namely “avoidant coping” and “approach coping” in the Brief-COPE. As the humor and religion subscales did not exclusively load on either of the aforementioned factors, they were not included in either. Avoidant coping is described by the Brief-COPE subscales of denial, substance use, venting, behavioral disengagement, self-distraction, and self-blame. It is not ideal at managing anxiety and has been linked with poorer physical health among those with medical conditions.^16^ On the other hand, approach coping is characterized by the subscales of active coping, positive reframing, planning, acceptance, seeking emotional support, and seeking informational support. Compared with avoidant coping, it has been associated with better responses to adversity, including adaptive practical adjustment, better physical health outcomes, and more stable emotional responding. However, Meyer categorized the strategies measured by the Brief-COPE into maladaptive coping and adaptive coping.^17^ Of these subscales, religion as well as humor were also considered as adaptive coping. Religious coping is defined as “religiously framed cognitive, emotional, or behavioral responses to stress, encompassing multiple methods and purposes as well as positive and negative dimensions”.^18^ In the present study, approximately 92% (doing this a lot = 56.9%; a moderate amount = 20.8%; a little bit = 14.2%) reported they have been trying to find comfort in their religion or spiritual beliefs, and 93.3% (doing this a lot = 47.8%; a moderate amount = 29.5%; a little bit = 16%) reported that they have been praying or meditating. It is interesting to note that scores for positive/adaptive coping strategies were greater than avoidant or maladaptive coping in our respondents.

This study had some limitations. First, this study was conducted among the students of 4 higher education institutions. Second, as this was a Web-based survey, the problem of selective participation and coverage error might be present. Third, we used a self-administered questionnaire, so disadvantages associated with self-report data (introspective ability, response bias, sampling bias) could exist. Fourth, in this study, there were no alpha adjustments made as this is an explanatory pilot study and no need of hypotheses. Last, the clinical assessment for the diagnosis of depression and anxiety disorders as per criteria of Diagnostic and Statistical Manual of Mental Disorders (DSM-V) was not done. However, our findings provide valuable insight about the psychological impact of COVID-19 among university students in Pakistan.

## CONCLUSIONS

The COVID-19 pandemic has a significant adverse impact on the mental health of Pakistani university students; prevalence of moderate to severe anxiety and depression were 34% and 45%, respectively. Major coping strategies adopted by the students are religious and acceptance coping. Our findings highlight that mental health should not be neglected during the epidemics. Educational institutions should work together with the authorities to promote measures suggested by the World Health Organization to keep mental health of their students in check.
